# From knife to needle – the trend of vascular neurosurgery in Belgium

**DOI:** 10.1016/j.bas.2024.104158

**Published:** 2024-12-11

**Authors:** Jorn Van Der Veken, Vera Van Velthoven, Gilles Reuter, Steven De Vleeschouwer, Tomas Menovsky, Johnny Duerinck, Michaël Bruneau

**Affiliations:** a Department of Neurosurgery, Flinders Medical Centre, Adelaide, South Australia, Australia; b Department of Neurosurgery, Universitair Ziekenhuis Brussel (UZ Brussel) - Vrije Universiteit Brussel (VUB), Laarbeeklaan 101-103, 1090, Brussels, Belgium; c Department of Neurosurgery, University Hospital of Liège (CHU de Liège), Liège, Belgium; d Department of Neurosurgery, University Hospital of Leuven (UZ Leuven), Leuven, Belgium; e Department of Neurosurgery, Antwerp University Hospital (UZA), Edegem, Belgium

**Keywords:** Cerebrovascular, Neurosurgery, Belgium, Aneurysm

## Abstract

**Introduction:**

The management of neurovascular pathologies has changed globally over the last few decades. Endovascular treatments are increasing, and fewer surgical procedures are performed.

**Research question:**

Evaluate the evolution of vascular neurosurgery in Belgium over the last 30 years and compare with other countries.

**Material and methods:**

Belgian nationwide data was obtained from 1991 to 2021 via the National Institute for Health and Disability Insurance (INAMI-RIZIV). Cost of surgical and endovascular implants used in 2022 was obtained.

**Results:**

Over a 30-year period a total of 37,504 procedures were performed in Belgium, consisting of 13,767 (36.7%) surgeries and 23,737 (63.3%) endovascular treatments (EVT).Adjusted to population per 100000, surgical management peaked at 6.02 in 1996 and gradually dropped below 3.0 in 2019. EVT increased from 1.06 in 1991 to 10.5 in 2021.Important regional differences are seen in terms of total number of procedures as well as ratio of surgery to endovascular surgery.The total cost of surgical implants in 2022 was the equivalent of 1% of the total cost of endovascular implants.

**Discussion and conclusions:**

This data confirms a similar trend to other regions in the world: a reduction in surgical management and increase in endovascular management for CNS vascular pathologies.Important regional differences are noted in terms of volume and ratio of endovascular to surgery.A nationwide registry based on patient data and outcomes can help decide whether there should be centralization to manage vascular pathologies.

## Highlights

• Less surgery is performed, whilst endovascular treatments are disproportionately increasing.
• The current cost of surgical implants is approximately equivalent to 1% of endovascular implants.
• Centralization for cerebrovascular surgery in Belgium should be considered to guarantee sufficient expertise.

## 1 Introduction

CNS vascular pathologies such as aneurysms, arteriovenous malformations and arteriovenous fistulas were traditionally treated surgically. A gradual shift away from surgery towards endovascular procedures appeared after the introduction of the Guglielmi coil in 1991 (Guglielmi et al., 1991; Dovey et al., 2001). Landmark trials such as ISAT and BRAT, paved the way for an increased use of endovascular treatments (McDougall et al., 2012; Molyneux et al., 2005). Additionally, in recent years a plethora of new endovascular tools appeared (stents, flow diverters, WEB devices, balloon-assisted coiling, hydrocoils), broadening the scope of pathology that can be treated endovascularly (Lee et al., 2022).

Geographical and regional differences have been reported, with centers adhering more to surgery or endovascular treatments depending on the experience or available facilities (Uhl et al., 2022; Frechon et al., 2021; Donnelly et al., 2022; Salem et al., 2021a). Due to an increasing pressure on healthcare budgets, cost-effectiveness of treatment has also become a more core consideration (Goertz et al., 2023; Twitchell et al., 2018).

In recent years a decline in surgical interventions for cerebrovascular pathologies has been witnessed by the authors. The goal of this study was to evaluate the evolution of vascular neurosurgery in Belgium over the last 30 years and what can be anticipated if this evolution continues. We also assessed regional differences and compared costs of different managements. This data should spark a discussion about centralized care of certain cerebrovascular conditions.

## 2 Methods

Belgian nationwide data was obtained from 1991 to 2021 via the National Institute for Health and Disability Insurance (INAMI-RIZIV). The INAMI-RIZIV is the federal public body of social security in Belgium, data can be provided on request. In the absence of a nationwide prospective registry, analysis was done based on retrospective data.

Included nomenclature, translated from the original Flemish and French version, is provided as supplemental data.

Calculation of treatment per 100000 people per year was based on the annual demographics as provided by statbel.fgov.be, an open access site managed by the Belgian government.

Regional data were analysed based on the data collected from the Brussels Capital Region and the 10 Belgian provinces. No regional data for the codes 232735–232746 were available.

## 3 Results

### 3.1 Number of interventions

Over a 30-year period a total of 37,504 procedures were performed in Belgium. 13,767 (36.7%) surgeries were performed and 23,737 (63.7%) endovascular treatments (EVT).

**Fig. 1:**
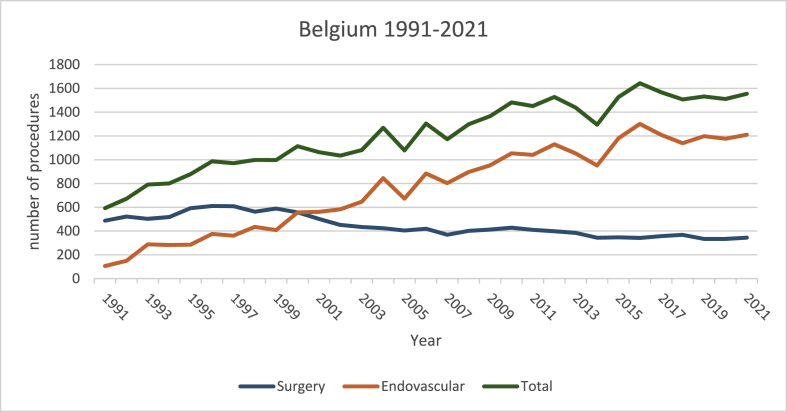
Graph showing the total number of endovascular and surgical procedures for CNS vascular pathologies in Belgium from 1991 till 2021.

In 1991 a total of 593 (487 surgeries, 106 EVT's) procedures were performed annually, which increased to 1063 in 2001 (502 surgeries, 561 EVT's), in 2011 to 1451 (410 surgeries, 1041 EVT's) and in 2021 to 1555 procedures annually (345 surgeries, 1210 EVT's) (Fig. 1).

**Fig. 2:**
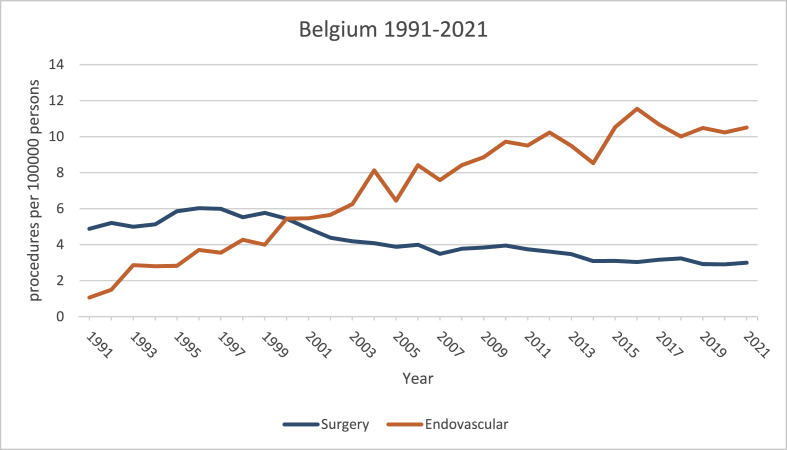
Number of procedures adjusted to population.

Adjusted to population per 100000, the number of surgeries peaked at 6.02 in 1996 and declined to 2.99 in 2021. EVT rose from 1.06 in 1991 to 10.5 in 2021 (Fig. 2).

### 3.2 Regional data

In 2021, 174 procedures were performed in Antwerp; 399 in the Brussels Capital Region, 148

In Hainaut; 166 in Limburg; 269 in Liège; 16 in Luxemburg; 37 in Namur; 180 in East Flanders; 99 in.

**Fig. 3:**
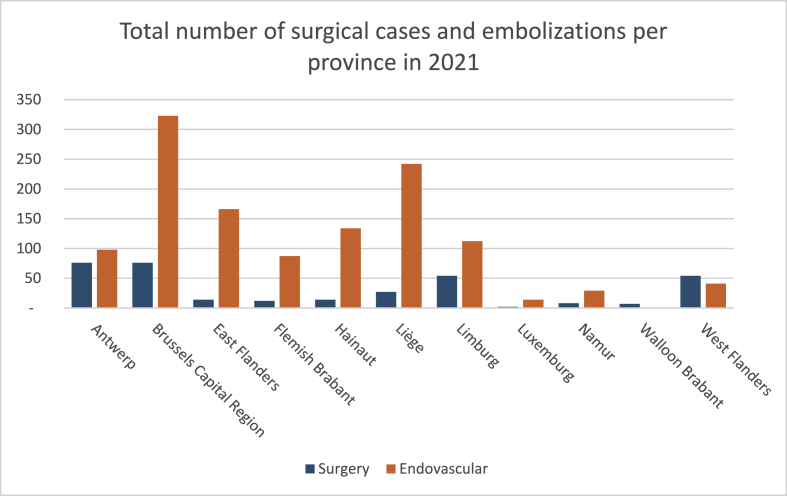
Total number of surgical and endovascular cases in 2021 per province.

Flemish Brabant; 7 in Walloon Brabant and 95 in West-Flanders. (Fig. 3).

Among these, 76 surgical cases were performed in Antwerp and Brussels Capital Region; 14 in Hainaut; 54 in Limburg; 27 in Liège; 2 in Luxemburg; 8 in Namur; 14 in East Flanders; 12 in Flemish Brabant; 7 in Walloon Brabant and 54 in West-Flanders. (Fig. 3).

A total of 98 embolizations were performed in Antwerp; 323 in Brussels Capital Region; 134 in Hainaut; 112 in Limburg; 242 in Liège; 14 in Luxemburg; 29 in Namur; 166 in East Flanders; 87 in Flemish Brabant; 0 in Walloon Brabant and 41 in West Flanders.

Therefore, the ratio between the number of surgical versus endovascular procedures was 43.7/56.3% in Antwerp; 19%/81% in Brussels Capital Region; 9.5%/90.5%in Hainaut; 32.5%/67.5% in Limburg; 10%/90% in Liège; 12.5%/87.5% in Luxemburg; 21.6%/78.4% in Namur; 77.8%/22.2% in East Flanders; 12.1%/87.9% in Flemish Brabant; 100%/0% in Walloon Brabant and 56.9%/43.1% in West-Flanders.

### 3.3 Fees per item number

The average fee per surgical item number (231011–231022 and 232551–232562) was 1584.76€ in 1991 versus 417.54€ for embolization (589116–589120; material excluded).

**Figure 4:**
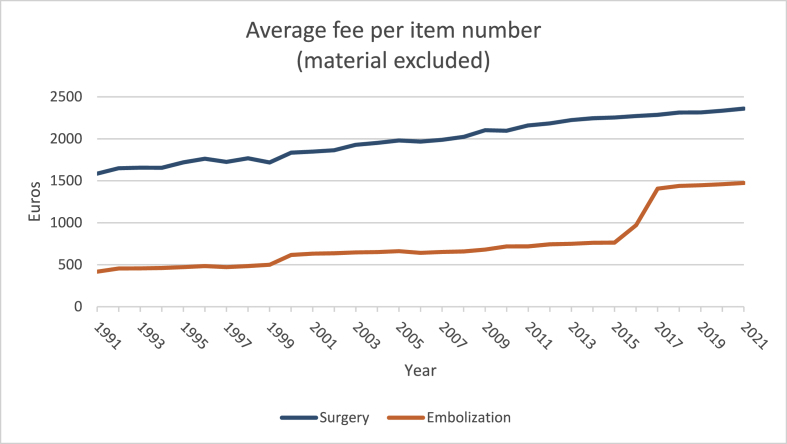
Average fee per item number per year for both surgery and embolization.

In 2001 this was 1847.35€ versus 631.04€ for embolization. In 2011, this became 2053.66€ for surgery versus 709.56€ for embolization, and in 2021, surgery was 2359.59€ and 1473.38€ for embolization). Hospitalization and material not included. (Fig. 4).

### 3.4 Cost of implants

**Table 1:** Overview of the implants used in open cerebrovascular^b^surgery in 2022.

Code^a^	Description^b^	Quantity	Cost (€)
152600	Aneurysm clips	328	75450
152025	Synthetic Dural Graft	826	4329
152622	Acrylic Cement (/10g)	56	3331
152644	Apatite Cement (/5g)	208	45371
152666	Cranial fixation (rivet)	145	7908
152681	Cranial fixation (rivet), large	2	149
152703	Burr hole cover	56	3609
152740	Cranial plate	394	17578
**Total**			157725

a codes in combination with nomenclature 232735 - 232746, 231011 - 231022 and 232551–232562.

b full description available on https://webappsa.riziv-inami.fgov.be/simpl/.

**Table 2:** Overview of the implants used for embolization in 2022.

Code^a^	Description^b^	Quantity	Cost (€)
174020	Set of basic catheterization equipment	866	243346
174042	Catheterization material	683	651582
174064	First microcatheter for embolization	1061	1357019
174086	Additional microcatheter	625	582500
174101	First microcatheter for embolization with solidifying material	159	217035
174123	Per additional microcatheter	153	165240
174145	Single lumen remodelling balloon	82	104632
174160	Double lumen remodelling balloon	324	585144
174182	Detachable coil	5501	6491180
174204	Stent to support embolization material in aneurysm	254	1016000
174226	Stent at bifurcation	8	72000
174241	Flow diverter/disruptor	268	3725200
174263	Additional radiopaque material	331	281350
174285	Microparticles for embolization	19	2945
174300	Thrombus retriever for evacuation of thrombus that appeared during embolization	15	66858
**Total**			15.562031

a Codes in combination with 589116–589120.

b Full description and details available on https://webappsa.riziv-inami.fgov.be/simpl/.

## 4 Discussion

### 4.1 Increase in total number of procedures

From 1991 to 2021, the total number of procedures increased from 593 to 1555 procedures annually (Fig. 1). Per 100000 this number increased from 5.8 to 13.3 (Fig. 2).

The available data do not differentiate between different vascular pathologies. There is a separate code for an EC-IC bypass; but only 246 bypasses were performed over a 30-year period (1992–2021).

We can, based on prevalence, assume that the majority of treatments are aneurysm treatments (Linfante and Wakhloo, 2007).

Why the number of treatments has tripled is not completely understood.

The prevalence of aneurysms among adults in Central European countries is about 3.2% (95% confidence interval) (M Vlak et al., 2011). With the more widespread use and better quality of imaging more vascular lesions are detected, but insufficient to account for the almost tripled number of lesions treated (Lee et al., 2021). The prevalence of AVMs is 10- to 15-fold lower than aneurysms (in the range of 0.2%), while AVFs are even more rare (Laakso and Hernesniemi, 2012). Unlike aneurysms, the detection rate has not increased (Gabriel et al., 2010).

As disease prevalence is insufficient to explain this trend; a lower threshold to treat is a more likely explanation (Khorasanizadeh et al., 2023). A trend of treating smaller aneurysms has been clearly documented in the United States, with a 0.71-mm decrease in the average size of treated UIAs every 5 years since 1987 (Khorasanizadeh et al., 2023). Some of the reasons for this paradigm shift are a better understanding of the natural history, improved and wider availability of imaging techniques, as well as safer and increased availability of endovascular treatment options (Khorasanizadeh et al., 2023). A higher retreatment rate with endovascular modalities is a likely contributing factor too. A recent systematic review looked at reopening and retreatment rates after coiled aneurysm. Aneurysm reopening occurred in 20.8% (95% CI, 19.8%–21.9%) and retreatment was performed in 10.3% (95% CI, 9.5%–11.0%) (Ferns et al., 2009). For surgery, retreatment rates are around 4–5% (Jabbarli et al., 2016; Spetzler et al., 2015).

### 4.2 Regional differences

The management of CNS vascular pathologies has seen a worldwide change, from an initial purely surgical approach to an era where endovascular management has become the preferred treatment modality. The pace at which this transition happened has been different in different countries and institutions (Uhl et al., 2022; Frechon et al., 2021; Donnelly et al., 2022; Khorasanizadeh et al., 2023; Bradac et al., 2012; Haverkamp et al., 2023; Golnari et al., 2021).

In Australia in 2008, more than half of all aneurysms were still being clipped (53.47%.) This number gradually dropped and in 2018 the favoured treatment modality became EVT with 63.49% (Donnelly et al., 2022).

In the US, in 2004 endovascular procedures were performed in around 52% of unruptured aneurysm procedures which increased to 73% by the end of 2014 (Salem et al., 2021b).

In Europe similar trends have been observed (Bradac et al., 2012). In Germany between 2007 and 2019, 62.6% involved EVT, 35.52% clipping and 1.8% combined (Haverkamp et al., 2023). During that same period in Belgium the ratio of EVT versus surgery was 74.4% versus 25.6% (all cerebrovascular pathologies). In France, known for its strong endovascular culture; in 2018, 80.5% off all aneurysms were treated endovascularly (75% of all unruptured and 85% of all ruptured aneurysms) (Frechon et al., 2021).

Within Belgium, clear differences are noted too. In terms of volume as well as ratio of surgery to endovascular, we see a wide variety between the different Belgian provinces (Fig. 3).

In 2021, the majority of surgical cases (77%) were performed in 4 provinces, Antwerp, Brussels Capital Region, Limburg and West-Flanders. Remarkable is the difference of treatments per 100000 inhabitants. In Brussels Capital Regions, this is 32/100000, in Antwerp 9.3, in Limburg 18.8 and in Western Flanders 7.9/100000. Important to note is that there are 3 different tertiary hospitals in Brussels Capital Region. It is possible they are referred patients from other provinces.

The oldest available data are from 1999, at which point still 7 out of 11 provinces performed more than 50 surgical cases a year, in 2021 only 4.

When comparing the ratio of surgery versus endovascular in Antwerp this is about 50/50, whereas in other regions such as East Flanders or Liège about a tenfold of EVT occur per surgery. Furthermore in some provinces less than 10 surgical and/or endovascular cases a year are performed.

### 4.3 Rising cost

Calculating and comparing the total cost of a surgical versus an endovascular procedure is complex. For an aneurysm where there is a therapeutic equipoise, the total cost for coiling is generally higher (Twitchell et al., 2018).

The **treatment** fee for endovascular embolization has tripled over the last 30 years (252.9%). The fee for surgical treatments has increased with 48.9% (Fig. 4).

In 1991, a total of 798604€ was spent on treatment fees (754345€ for surgery, 44259€ for ETV). In 2021 this increased to 2568532€ (785742€ for surgery and 1782789 for ETV).

However, this data does not take into account the cost of the materials used, which implies the largest cost (Goertz et al., 2023; Twitchell et al., 2018).

In 2022, the total **cost of the devices** used for endovascular occlusion of a vascular pathology in the brain or spinal cord, was 15.579607€ (breakdown of largest contributors in Table 2). The most important contributors were 268 flow diverters (13900 € each), 254 Stents (4000 € or 9000 € each) and 5591 Coils (1180 € each). Whereas the cost of a surgical clip is 230,03 € each. Table 1 gives an overview of the costs of the implants used when performing a surgical clipping or excision. Overall, the cost of surgical implants is the equivalent of about 1% of the cost of endovascular implants.

For a full economic assessment, the cost of the hospital stay, complications, time off work, rehabilitation and retreatment rates should be included, but this data is currently not available (Lad et al., 2013).

To determine which treatment strategy is most cost-effective, more prospective data is needed.

### 4.4 Implications for the future

Belgium has seen a spectacular rise in number of endovascular procedures performed for vascular CNS pathologies, in line with other countries worldwide (Graffeo et al., 2022). However current available data are lacking details for in depth analysis.Table 2

To have a better understanding of the incidence of CNS vascular pathologies, the associated morbidity and mortality of treatments; evaluate the cost and assess quality and quantity of care in Belgian centers, we would advocate for a nationwide database.

Qermid (Quality Electronic Registration of Medical acts, Implants and Devices) has recently been introduced for spinal procedures, spinal cord stimulators, as well as non-neurosurgical specialties such as cardiac surgery and orthopaedics (Hanet et al., 2018).

A more diversified nomenclature, subdividing for aneurysms, AVMs and fistulas, retreatment etc. would also facilitate data interpretation. In 2022 a new nomenclature was for the treatment of spinal arteriovenous fistulas and malformations (226273-226282), but no other changes have been implemented since. Registration of patient specific data (size, location, cerebrovascular risk factors, complications, retreatment rates) would lead to optimized and more cost-efficient care.

The importance of sufficient exposure of neurosurgical trainees to open cerebrovascular surgery cannot be overemphasized (Graffeo et al., 2022; Huang et al., 2023). Experience is necessary to reassure adequate surgical competency and clinical judgement (Huang et al., 2023). The current Belgian accreditation requirements do not demand a minimum number of open cerebrovascular cases, nonetheless most neurosurgeons manage patients with vascular pathologies.

Ideally patients with CNS vascular pathologies should be managed by a multidisciplinary team, in order to tailor the best treatment strategy to the individual patient. Numerous studies have shown significant differences in morbidity and mortality in highvolume centers with high-volume surgeons compared with low-volume surgeons and hospitals for the management of cerebrovascular diseases (Connolly et al., 2006). Also from an economical perspective, management of cerebrovascular diseases is safer less expensive at regional medical centers with specialized facilities and medical personnel (Connolly et al., 2006). With 68 neurosurgical centers in Belgium, of which few have neurosurgeons with subspecialty training in cerebrovascular surgery, it seems questionable whether patients are receiving optimal care. For the above-mentioned reasons, centralization needs to be considered.

### 4.5 Limitations

There are some clear limitations with this data. Firstly, they are derived from a financial database and do not provide patient information. The nomenclature does not differentiate between aneurysms, fistulas or AVMs. Nor does it differentiate between cranial or spinal pathologies, or elective versus emergency. Limitations regarding the cost evaluation were already discussed.

## 5 Conclusion

Over the last 30 years a gradual decrease of surgery for neurovascular pathologies has been observed. There has been a disproportionate rise in endovascular treatments, with currently a threefold of endovascular treatments for every surgery.

From a quality, epidemiological and economical perspective, we would advocate for the introduction of a nationwide registry.

Centralised care of vascular CNS pathologies should be considered.

## Credit author statement

Jorn Van Der Veken: Conceptualization, Methodology, Writing, Investigation.

Van Velthoven: Supervision, Writing - Review & Editing.

Gilles Reuter: Review & Editing.

Tomas Menovsky: Review & Editing.

Steven De Vleeschouwer: Review & Editing.

Johnny Duerinck: Supervision, Writing - Review & Editing.

Michaël Bruneau: Supervision, Writing - Review & Editing.

## Declaration of competing interest

This research did not receive any specific grant from funding agencies in the public, commercial, or not-for-profit sectors.

The authors declare no conflicts of interest.

Generative AI was not used for this manuscript.
