# Supplementary material for: From knife to needle – the trend of vascular neurosurgery in Belgium
Source: Brain Spine. 2024 Dec 11;5:104158. doi: 10.1016/j.bas.2024.104158 (PMC11719377; doi:10.1016/j.bas.2024.104158)
Supplement: Multimedia component 1 [file mmc1.docx]

Surgical:

**231011 – 231022**, created on 1/07/1986: Surgical treatment of an intracranial aneurysm or angioma via trepanation.

**232551 – 232562**, created on 1/08/1988: Aneurysm or arteriovenous connection: Surgical treatment of an arteriovenous connection of the brain, spinal cord or meninges via trepanation or laminectomy

**232735 – 232746**, created on 1/08/1988: Extracranial to intracranial vascular anastomosis by microsurgical technique

Endovascular:

**589116 – 589120,** created on 01/01/1991: Percutaneous occlusion under radiological control of the arterial or venous vascularization of 1 or more pathological lesion in the brain or spinal cord by physical or chemical products; included are: manipulations, controls, catheters; excluded are embolization catheters, pharmaceuticals, contrast and embolization material

Codes of implants used to calculate costs: 152014, 152025, 152036, 152040, 152596, 152600, 152611, 152622, 152633, 152644, 152655, 152666, 152670, 152681, 152692, 152703, 152714, 152725, 152736, 152740. Full description publicly available on <https://webappsa.riziv-inami.fgov.be/simpl/>.

Supplementary material: details of the different UNAMI-RIZIV codes used in this study
